# Significantly Improved Recovery of Recombinant Sonchus Yellow Net Rhabdovirus by Expressing the Negative-Strand Genomic RNA

**DOI:** 10.3390/v12121459

**Published:** 2020-12-17

**Authors:** Xiaonan Ma, Zhenghe Li

**Affiliations:** 1 State Key Laboratory of Rice Biology, Institute of Biotechnology, Zhejiang University, Hangzhou 310058, China; xiaonma@foxmail.com; 2 Ministry of Agriculture Key Laboratory of Molecular Biology of Crop Pathogens and Insect Pests, Zhejiang University, Hangzhou 310058, China; 3 Key Laboratory of Biology of Crop Pathogens and Insects of Zhejiang Province, Zhejiang University, Hangzhou 310058, China

**Keywords:** negative-stranded RNA virus, plant rhabdovirus, Sonchus yellow net virus, recovery, infectious clone, viral suppressor of RNA silencing, genome, antigenome, nucleocapsid, matrix protein

## Abstract

Generation of recombinant negative-stranded RNA viruses (NSVs) from plasmids involves in vivo reconstitution of biologically active nucleocapsids and faces a unique antisense problem where the negative-sense viral genomic RNAs can hybridize to viral messenger RNAs. To overcome this problem, a positive-sense RNA approach has been devised through expression of viral antigenomic (ag)RNA and core proteins for assembly of antigenomic nucleocapsids. Although this detour strategy works for many NSVs, the process is still inefficient. Using Sonchus yellow net rhabdovirus (SYNV) as a model; here, we develop a negative-sense genomic RNA-based approach that increased rescue efficiency by two orders of magnitude compared to the conventional agRNA approach. The system relied on suppression of double-stranded RNA induced antiviral responses by co-expression of plant viruses-encoded RNA silencing suppressors or animal viruses-encoded double-stranded RNA antagonists. With the improved approach, we were able to recover a highly attenuated SYNV mutant with a deletion in the matrix protein gene which otherwise could not be rescued via the agRNA approach. Reverse genetics analyses of the generated mutant virus provided insights into SYNV virion assembly and morphogenesis. This approach may potentially be applicable to other NSVs of plants or animals.

## 1. Introduction

Virions of negative-stranded RNA viruses (NSVs) must enclose not only the negative-strand genomes but also nucleocapsid (NC) core proteins, because the naked genomic (g)RNAs alone are unable to initiate translation of viral proteins and genome replication. Rather, the minimal infectious unit of an NSV is an NC or ribonucleoprotein (RNP) complex consisting of the nucleoprotein (N) that tightly encapsidates the genome along its entire length, the large RNA-dependent RNA polymerase (L) and its non-catalytic co-factor, e.g., the phosphoprotein (P) in the case of rhabdoviruses (family *Rhabdoviridae*). Upon entry into a host cell, the genomic (g)NC is released from virion, and the viral polymerase complex immediately carries out messenger (m)RNA transcription using the encapsidated genome as a template. The de novo synthesized viral proteins are required for initiation of replication to produce antigenomic (ag)RNAs that are also concomitantly encapsidated by the N proteins to form antigenomic (ag)NCs. Following cyclic waves of mRNA transcription and genome replication, virion assembly and budding occur to produce progeny virus particles ready for new rounds of infection in neighboring cells [1,2].

As the infection cycles of NSVs dictate, generation of recombinant viruses from cloned complementary DNAs (cDNAs) necessitates in vivo reconstitution of biologically active RNPs from individual components, i.e., viral RNA and NC core proteins. However, early attempts to recover infectious viruses by reconstituting gNCs through co-expression of gRNAs and core proteins have repeatedly failed [3,4,5,6,7,8]. A turning point occurred in 1994 when Schnell and colleagues decided to express an agRNA rather than the gRNA of rabies virus (a mammalian rhabdovirus), and then the recombinant virus was recovered [7]. Use of agRNA is believed to be the key to success because it avoids an antisense problem associated with NSV rescue experiments. If the negative-strand gRNAs are used, the core proteins–encoding mRNAs could hybridize to the gRNAs. Formation of double-stranded (ds)RNAs not only hampers the template activities of the mRNA for translation but also prevents gRNA encapsidation [6,8,9]. Additionally, dsRNA is considered to be a pathogen-associated molecular pattern that triggers potent antiviral immunity across eukaryotic kingdoms, i.e., the interferon response in vertebrates [10] and RNA interference (RNAi) in plants, fungi, insects, and nematodes [11]. Nevertheless, since the initial success with rabies virus, this positive-stranded agRNA approach was quickly applied to other nonsegmented vertebrate NSVs (order *Mononegavirales*), leading to successful rescues of rhabdoviruses [3,4], paramyxoviruses [5,12,13,14,15], filoviruses [16], and bornaviruses [17]. Moreover, similar strategies have proven suitable for recovery of vertebrate NSVs with segmented genomes, such as the bi-segmented arenaviruses [18], the tri-segmented bunyaviruses [19,20,21], and phleboviruses [22,23,24]. In rare cases, recombinant NSVs have also reportedly been generated by using the negative-strand gRNAs, but the efficiencies were substantially lower than those by using their agRNAs [15,25].

Reverse genetics analyses of plant NSVs have lagged behind the vertebrate counterparts owing to a number of technical difficulties in addition to the inherent low efficiencies of RNP assembly [26]. These technical hurdles were partially circumvented recently by using Sonchus yellow net rhabdovirus (SYNV) as a model. Recombinant SYNV was successfully recovered in *Nicotiana benthamiana* plants after *Agrobacterium*-mediated delivery of binary plasmids for co-expression of the SYNV agRNA, the N, P, and L core proteins, and viral suppressors of RNA silencing (VSRs) [27]. Similar strategies were applicable to a barley yellow striate mosaic cytorhabdovirus, although infections of its native monocot hosts require insect vector-mediated transfer of the recombinant virus recovered from *N. benthamiana* leaves [28]. Recently, refined positive-strand agRNA strategies were also adapted to rescue a tri-segmented tospovirus [29] and a seven-segmented emaravirus [30]. In these two cases, expression of viral core proteins (N and L) from in trans provided supporting plasmids is unnecessary since the agRNA transcripts permit direct ribosomal translation to produce both core proteins in cis [29,30].

Although refined techniques based on the agRNA approach appeared to be generally applicable to members of the major animal and plant NSV families, these processes are still inefficient. For example, Schnell et al. reported the generation of recombinant rabies virus in only ~1 of 10^7^ transfected cells [7]. Likewise, the initial SYNV rescue protocol resulted in only sporadic infection foci in agroinfiltrated leaves and as few as 5% of agroinoculated plants eventually developed systemic infection [27]. In this study, we showed that the expression of genome-sense RNA templates and VSRs for RNAi suppression drastically increased the rescue efficiency. The improved rescue system achieved up to a 100% systemic infection rate in agroinfiltrated plants and permitted recovery of a highly attenuated SYNV mutant.

## 2. Materials and Methods

### 2.1. Construction of Plasmids

The plasmids pSYNV-GFP(+) and pSYNV-GFP-M:RFP(+) used for agRNA transcription have been described previously [27]. To construct the plasmids pSYNV-GFP(-) and pSYNV-GFP-M:RFP(-) for intracellular transcription of the gRNA derivatives, we amplify the viral cDNA sequence from the pSYNV-GFP(+) and pSYNV-GFP-M:RFP(+) plasmids by PCR using the forward primer SYNV/ trailer/F (5’-AGAGACAAAAGCTCAGAACAATCCCTAT-3’) and reverse primer SYNV/ leader/R (5’-AGAGACAGAAACTCAGAAAATACAATCACCGT-3’). At the meantime, the pCB301-HDV binary plasmid [27] was linearized by PCR with the primers pCB301/35s/F (5’-TGAGCTTTTGTCTCTCCTCTCCAAATGAAATGAAC-3’) and pCB301/HDV/R (5’-TGAGTTTCTGTCTCTGGGTCGGCATGGCAT-3’). The viral cDNA fragments were separately ligated with the linear plasmid fragment by using an In-Fusion HD PCR Cloning kit (Clontech, Japan), such that the inserts were positioned in an inverted orientation relative to those in the pSYNV-GFP(+) and pSYNV-GFP-M:RFP(+) plasmids.

The pGD-NPL plasmid for tandem expression of the SYNV N, P, and L proteins, and the pGD-HcPro, pGD-p19, and pGD-γb plasmids for expression of tobacco etch virus (TEV) P1/HcPro, tomato bushy stunt virus (TBSV) p19, and barley stripe mosaic virus (BSMV) γb, respectively, have been described previously [27,31]. To generate binary vectors for expression of animal virus-encoded dsRNA-binding proteins, the coding sequences of flock house virus B2, Ebola virus VP35, and influenza A virus-H1N1 NS1 protein (GenBank accession nos. X77156, AF086833, and J02150, respectively) were chemically synthesized by GenScript (Nanjing, China) and subcloned into the pGD vector [32].

### 2.2. Agrobacterium Infiltration

Recombinant binary plasmids were introduced into *Agrobacterium* (EHA105) by electroporation. Overnight cultures of *Agrobacterium* cells were resuspended in infiltration buffer (10 mM MES, 10 mM MgCl_2_, and 100 μM Acetosyringone, pH 5.6) at an optical density OD_600_ of 0.6 and activated for 2–4 h at room temperature. Before infiltration, *Agrobacterium* cultures harboring the gRNA or agRNA transcription plasmid were mixed with the cultures containing the pGD-NPL plasmid at a 1:1 ratio unless otherwise stated, supplemented with one volume of bacterial mixtures containing the VSRs. The mixed cultures were infiltrated into leaves of four-week-old *N. benthamiana* plants using a 1-mL syringe. Infiltrated plants were grown in a greenhouse at 25 °C with a 16-h light/8-h dark photoperiod.

### 2.3. Immunoblotting

Total proteins extracted from healthy or infected *N. benthamiana* leaves were separated by 12.5% SDS-polyacrylamide gel electrophoresis, transferred to nitrocellulose membranes, and probed with either polyclonal antibodies against disrupted SYNV virions [33] or monoclonal antibodies against GFP and RFP (Abcam, China).

### 2.4. Fluorescence Imaging

Fluorescence in infiltrated or systemically infected leaves was monitored with a Zeiss SteREO Lumar V12 epifluorescence microscope using the filter Lumar 38 for GFP detection (excitation, 470/40 nm; emission, 525/50 nm) and Lumar 31 for RFP detection (excitation, 565/30 nm; emission, 620/60 nm). *N. benthamiana* plants systemically infected with SYNV derivatives were also photographed under visible light and long-wavelength ultraviolet illumination.

### 2.5. Transmission Electron Microscopy and Immunogold Labeling

Leaf tissues systemically infected with SYNV-GFP or SYNV-GFP-M:RFP were excised, fixed, and embedded in Epon 812 resin as described previously [27]. The ultrathin sections (70 nm) were cut from the embedded tissues, stained with uranyl acetate for 10 min and with lead citrate for another 10 min, and then examined with a transmission electron microscope (TEM; H-7650, Hitachi, Japan).

For immunogold labeling assay, leaf samples were vacuumized and fixed in a 100 mM phosphate-buffered saline (PBS, pH 7.2) containing 3% formaldehyde and 0.1% glutaraldehyde for 3 h at 4 °C. The samples were subsequently dehydrated in graded ethanol solutions (30%, 50% at 4 °C (30 min each) and then 50%, 70%, 90%, 100%, 100%, 100% at –20 °C (1 h each)). The dehydrated samples were embedded in Lowicry K4M resin at −20°C for 72 h with UV radiation and then at room temperature for 48 h. After resin polymerization, ultrathin sections (70 nm) were cut from embedded tissue blocks and mounted onto nickel grids. Sections on grids were first blocked for 30 min by floating on drops of blocking buffer (50 mM PBS pH 7.0, 1% BSA, 0.02% polyethylene glycol 20000, 100 mM NaCl, and 0.1% NaN_3_), followed by incubation at room temperature for 2 h in 1:200 (*v*/*v*) diluted rabbit polyclonal antibodies against disrupted SYNV virions. The sections were washed several times with PBS and then incubated in 1:100 (*v*/*v*) diluted goat anti-rabbit IgG conjugated with gold particles for 1 h. Finally, the sections were rinsed in PBS and distilled water before staining with uranyl acetate and lead citrate, and examined under a transmission electron microscope (TEM; H-7650, Hitachi, Japan).

## 3. Results

### 3.1. Use of Genome-Sense RNA Template Improves SYNV Rescue Efficiency by Two Orders of Magnitude

**Figure 1:**
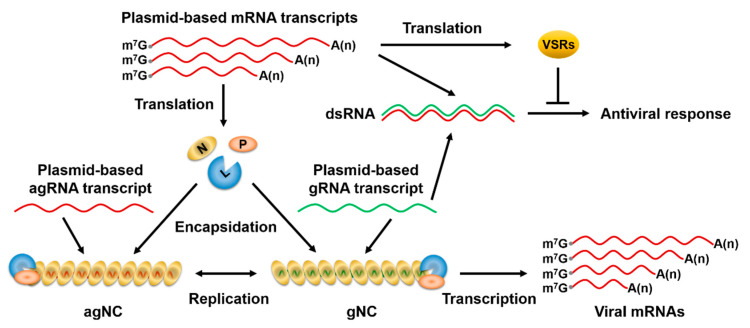
Positive- and negative-strand approaches for recovery of recombinant rhabdoviruses from cloned cDNAs. The positive-strand approach relies on plasmids-based co-expression of viral antigenomic (ag)RNA with the N, P, and L nucleocapsid (NC) core proteins to assemble the antigenomic (ag)NC, which serves as a replication template to produce genomic (g)RNA that is wrapped along its length by the NC proteins to form genomic (g)NC. In the negative-strand approach, although base-pairing between plasmids-derived viral mRNAs and gRNA transcripts results in double-stranded (ds)RNA formation, antiviral RNAi responses are minimized in the presence of co-expressed viral suppressors of RNA silencing (VSRs). Therefore, gNC can be directly reconstituted from viral gRNA and NC proteins to jump-start viral transcription, and subsequent infection steps occur as in natural virus infections. m^7^G, N^7^-methylguanosine cap; A(n), polyadenylated tail.

During natural NSV infections, the released gNC encompassing the negative-sense genome is the infectious entity that starts the infection cycles. However, in the conventional agRNA-based NSV reverse genetics systems, agNC is first assembled from co-expressed NC core proteins and agRNA, which must undergo a genome replication step to synthesize gRNA for gNC assembly. Once biologically active gNC is assembled, subsequent infection processes can ensue, with the gNC acting as a template for both genome replication and mRNA transcription. In theory, if one starts with expressing the gRNA template and NC proteins and meanwhile manages to avoid the antisense problem, gNC could be directly reconstituted to jump-start the infection cycles, bypassing the replication step from the agNC to gNC (Figure 1). We reason that in the presence of VSRs that function to boost transient expression and minimize RNA silencing in plants, a gRNA-based approach may outperform the agRNA approach since it more closely resembles the natural infection cycles.

**Figure 2:**
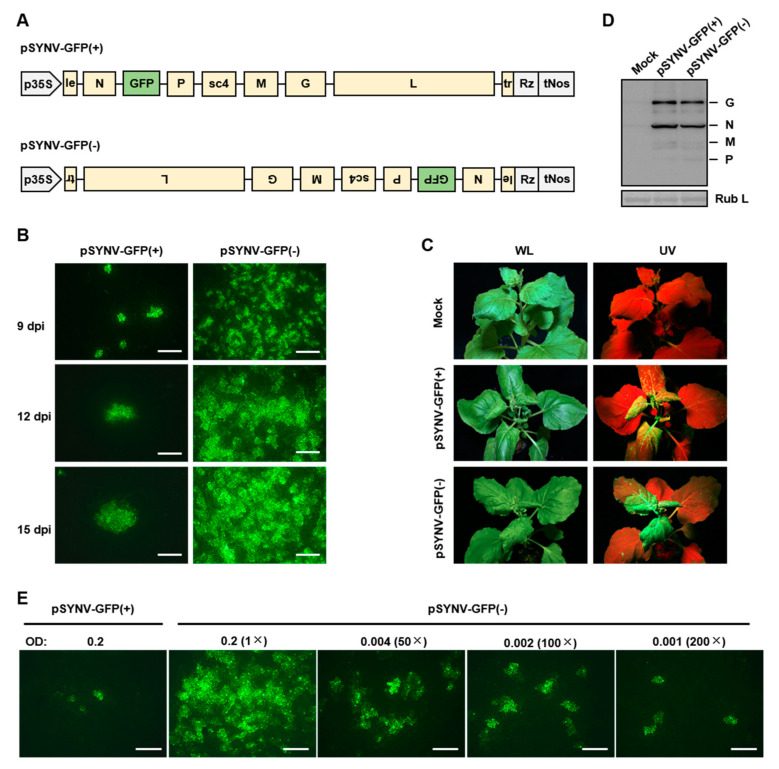
A genome-sense RNA approach drastically increased recombinant SYNV recovery. (**A**) Schematic representation of the pSYNV-GFP(+) and pSYNV-GFP(-) plasmids for transcription of the SYNV agRNA and gRNA derivatives containing an engineered GFP expression cassette. p35S, cauliflower mosaic virus doubled 35S promoter; le, leader; tr, trailer; Rz, hepatitis delta virus antigenomic ribozyme; tNos, Nos terminator. (**B**) GFP foci in *N. benthamiana* leaves infiltrated with agrobacterial mixtures harboring the pSYNV-GFP(+) or pSYNV-GFP(-), along with supporting plasmids designed for expression of SYNV core proteins and viral suppressors of RNA silencing. Infiltrated leaves were photographed at 9, 12, 15 days post infiltration (dpi) with a fluorescence microscope. (**C**) Symptoms elicited by recombinant SYNV-GFP recovered from pSYNV-GFP(+) at 30 dpi or pSYNV-GFP(-) at 20 dpi. Plants were photographed under white light (WL) and ultraviolet light (UV). (**D**) Western blot analysis of the expression of viral structural proteins (G, N, M, and P) in upper leaf tissues of systemically infected plants at 30 dpi. The large subunit of RuBisCO (Rub L) stained by Coomassie Brilliant was used as a loading control. (**E**) Comparison of the GFP foci numbers at 9 dpi in leaves infiltrated with the pSYNV-GFP(+) or pSYNV-GFP(-) agrobacterial cultures with different dilutions. Scale bars in (**B**) and (**E**) = 500 μm.

To test this hypothesis, we constructed the binary plasmid pSYNV-GFP(-) for transcription of a negative-sense SYNV gRNA derivative harboring a GFP reporter cassette inserted between the *N* and *P* genes to facilitate tracking of recombinant virus. This plasmid contained identical viral cDNA sequence but in an inverted orientation with the previously engineered agRNA transcription plasmid pSYNV-GFP(+) [27] (Figure 2A). *N. benthamiana* leaves were agroinfiltrated to deliver the pSYNV-GFP(-) or pSYNV-GFP(+), together with the pGD-NPL plasmid for expression of the N, P, and L core proteins, and the pGD plasmids designed for expression of the TEV HcPro, TBSV p19, BSMV γb VSRs. As shown previously [27], sporadic single-cell GFP foci indicative of recombinant virus recovery appeared in the pSYNV-GFP(+)-infiltrated leaves by nine days post infiltration (dpi). In contrast, numerous infection foci developed in the pSYNV-GFP(-)-infiltrated leaves (Figure 2B). Cell-to-cell movement of recombinant SYNV-GFP was apparent by 12 dpi and more extensive at 15 dpi. In the plants infiltrated with pSYNV-GFP(-), systemic infections began to develop on emerging leaves by 15 dpi; however, for the pSYNV-GFP(+)-infiltrated plants, disease onset was not observed until 22 dpi, at least a seven-day delay relative to the pSYNV-GFP(-) plants. Nevertheless, the recombinant viruses recovered from the agRNA or gRNA templates ultimately elicited indistinguishable symptoms and exhibited similar structural protein accumulations (Figure 2C,D).

To more precisely compare the rescue efficiencies, plants were infiltrated with the diluted pSYNV-GFP(-) cultures and the undiluted pSYNV-GFP(+) cultures, and the numbers of local infection foci and systemic infection rates were determined. Whereas the pSYNV-GFP(+) infiltrated leaves produced 4.3 foci per observation field, the undiluted, 50-, 100-, and 200-fold diluted pSYNV-GFP(-) cultures yielded over 100, 32.0, 18.3, and 5.3 foci per field, respectively (Figure 2E; Table 1). The local foci numbers roughly correlated with the systemic infection rates. Recombinant infections were detected in 100% of plants inoculated with the undiluted pSYNV-GFP(-). Even the 100- and 200-fold diluted culture resulted in 8.8% and 2.2% infected rates, which were comparable to the 6.7% for the pSYNV-GFP(+) cultures (Table 1). Together, our data show that the gRNA approach is about two orders of magnitude more efficient than the agRNA approach in the recovery of SYNV from cloned cDNA.

### 3.2. RNAi Suppression Is Essential for SYNV Recovery Using the gRNA Approach

**Figure 3:**
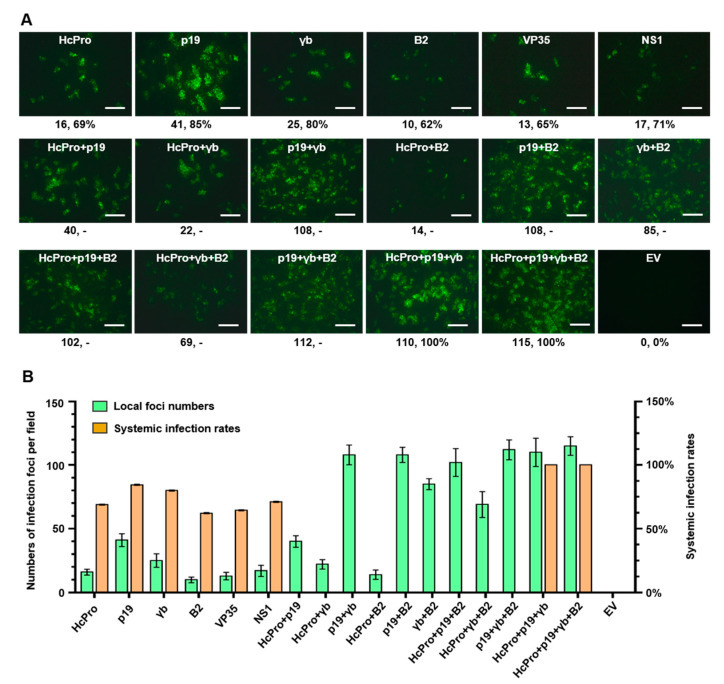
Effects of various co-expressed RNA silencing suppressors on SYNV rescue from a genomic cDNA clone. (**A**) Fluorescent microscope images showing the GFP foci in infiltrated leaves. *N. benthamiana* leaves were agroinfiltrated with bacterial cultures harboring the genomic cDNA clone pSYNV-GFP(-) and the supporting plasmids for expression of viral core proteins and a single or combinatory RNAi suppressors. GFP foci in agroinfiltrated leaf tissues were photographed with a fluorescence microscope at 9 dpi. The numbers (#, #%) below each panel are the average number of infection foci per field in infiltrated leaves at 9 dpi and the percentage of systemically infected plants per total agroinfiltrated plants at 30 dpi. Scale bar = 500 μm. (**B**) Bar chart summarizing the efficiencies of the local and systemic recovery of recombinant SYNV. Data are the means of three biological replicates for foci number counting and of three independent inoculation experiments for the calculation of systemic infection rates. Bars represent standard deviations. -, not determined. EV, empty binary vector.

We next tested the effect of VSRs on SYNV recovery from co-expressed gRNA and core proteins. As shown in Figure 3, expression of a single VSR—i.e., TEV HcPro, TBSV p19, and BSMV γb—resulted in 16–41 infection foci per field in agroinfiltrated leaves, with the p19 protein being the most efficient VSR (41 foci per field). However, no GFP focus was detected throughout the infiltrated leaf tissues when VSR was absent (EV), suggesting that suppression of RNAi is of critical importance for in vivo RNP assembly. As the gRNA approach is anticipated to produce viral dsRNAs, we also tested several animal viruses-encoded dsRNA binding proteins that function to antagonize dsRNA-induced RNAi and/or interferon response, including flock house virus B2 [34], Ebola virus VP35 [35], and influenza virus NS1 [36]. Expression of each of these proteins also facilitated SYNV rescue, although the resulting foci numbers (10–17 per filed) were slightly fewer than the leaves expressing the VSRs of plant viruses. Moreover, combinations of two, three, or four of these suppressors generally exhibited additive effects on foci number increases. Again, the combinations with the p19 protein displayed greater numbers of GFP foci in general than those without.

A large batch of the agroinfiltrated plants expressing individual suppressors were monitored for systemic infection. Infection rates ranging from 62–85% were detected in these plants, whereas the plants expressing three or four suppressors achieved 100% infection rates (Figure 3A,B). As anticipated, no plants developed a systemic infection when the VSR expression plasmid was omitted. Therefore, suppression of dsRNA-triggered RNAi response is essential for recombinant virus recovery.

### 3.3. Improved Rescue System Permits Recovery of a Highly Attenuated SYNV Mutant Virus

**Figure 4:**
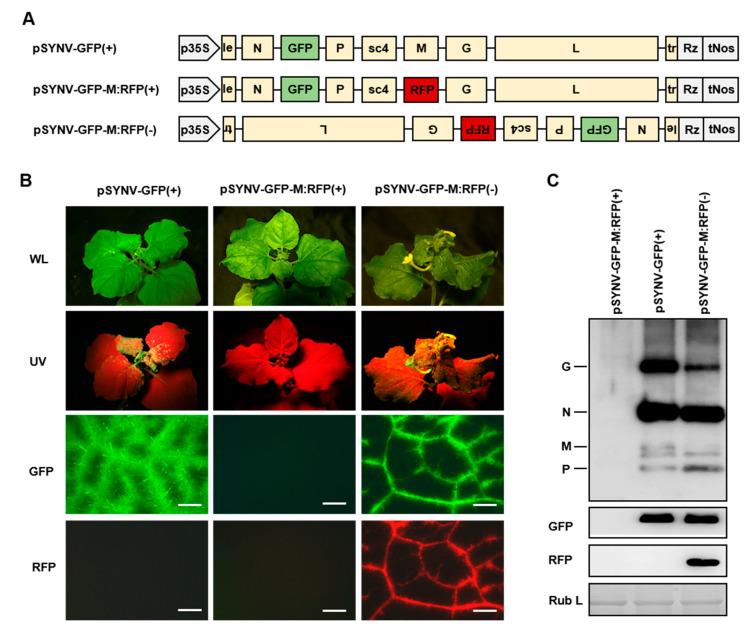
Recovery of recombinant SYNV M deletion mutant from a genomic but not antigenomic cDNA clone. (**A**) Schematic representation of the pSYNV-GFP-M:RFP(+) and pSYNV-GFP-M:RFP(-) clone designed for transcription of an antigenomic or genomic RNA in which the RFP gene was substituted for the M gene. (**B**) Symptoms and fluorescence in *N. benthamiana* plants agroinfiltrated to deliver the plasmid pSYNV-GFP(+), pSYNV-GFP-M:RFP(+), or pSYNV-GFP-M:RFP(-), together with supporting plasmids required for recombinant virus recovery. Plants were photographed under visible light or UV light at 10 days post systemic infection. Leaf tissues were also photographed with a fluorescence microscope to show the GFP and RFP expression patterns in the upper systemically infected leaves. Scale bar = 500 μm. (**C**) Protein gel blots showing the accumulations of viral proteins and reporter proteins in upper leaf tissue extracts. The Coomassie brilliant blue-stained large subunit of RuBisCO (Rub L) serves as a protein loading control.

Rhabdovirus matrix (M) proteins are required for virus assembly and budding and constitute major virion structural components [37]. Previously, we were unable to recover an SYNV M deletion mutant by using the positive-strand agRNA approach [27]. With the improved rescue system, we tested whether the SYNV M deletion mutant can be rescued from plasmids using the negative-strand gRNA approach. To this end, we generated the pSYNV-GFP-M:RFP(-) plasmid for transcription of an SYNV gRNA derivative with the M gene being replaced by a red fluorescence protein (RFP) gene (Figure 4A). Meanwhile, the pSYNV-GFP-M:RFP(+) plasmid designed for transcription of an agRNA equivalent was used as a control. As anticipated based on our previous study [27], none of the 45 plants agroincolated with the pSYNV-GFP-M:RFP(+) cultures was infected systemically. In contrast, about 9% of plants agroincolated with the pSYNV-GFP-M:RFP(-) cultures began to develop virus symptoms on newly emerged leaves starting from 40 dpi (Table 1; Figure 4B). Successful recovery of the recombinant SYNV-GFP-M:RFP by the gRNA approach was further confirmed by the presence of GFP and RFP fluorescence in systemically infected leaf tissues. Notably, unlike the GFP-tagged wild-type virus (SYNV-GFP) that had spread throughout the systemic leaf tissues, SYNV-GFP-M:RFP was mostly confined to the leaf veins as detected by fluorescence imaging (Figure 4B). Protein gel blot assays using SYNV virion antibodies verified the presence of N, P, and glycoprotein (G) proteins but the absence of M protein in upper leaf tissues of plant systemically infected with SYNV-GFP-M:RFP(-) (Figure 4C). Despite the limited mesophyll tissue distribution, SYNV-GFP-M:RFP infections yielded similar levels of the N and P structural proteins and the GFP reporter proteins to SYNV-GFP infections, but the accumulation of the membrane G protein were substantially reduced (Figure 4C). In conclusion, our data show that the SYNV M deletion mutant is viable but is highly attenuated in terms of systemic infectivity and tissue invasiveness.

**Figure 5:**
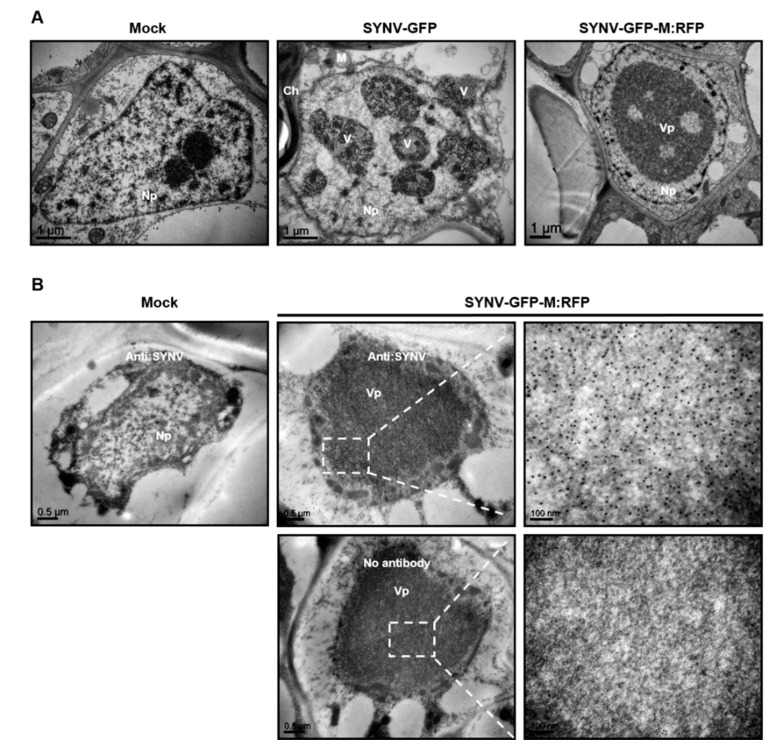
Cytopathology elicited by SYNV-GFP-M:RFP infections. (**A**) Electron micrographs of ultrathin sections of *N. benthamiana* plants mock infected, infected with SYNV-GFP or SYNV-GFP-M:RFP. (**B**) Representative electron micrographs showing immunogold-labeled viroplasms in SYNV-GFP-M:RFP infected nuclei. Ultrathin sections were first incubated in buffer with or without primary polyclonal antibodies raised against disrupted SYNV virion (anti-SYNV) and then with secondary anti-IgG antibody conjugated with gold particles. Boxed regions are enlarged on the right panels to highlight the distribution of gold particles. Ch: chloroplast; M: mitochondria; Np: nucleoplasm; V: virion; Vp: viroplasm.

To provide more insights into the roles of the M protein in SYNV infection and cytopathology, we used transmission electron microscopy to analyze virus-associated ultrastructure in upper leaf tissues infected with SYNV-GFP and SYNV-GFP-M:RFP. As shown in Figure 5A, large numbers of enveloped, bacilliform particles enclosed by invaginated nuclear envelopes were present in the nuclei of SYNV-GFP-infected cells. However, in the cells infected with SYNV-GFP-M:RFP, although electron-dense large inclusions characteristic of the subnuclear viroplasms were evident, no enveloped or nonenveloped virus particle was observed after examining multiple thin sections. The nature of these subnuclear inclusions were verified by immuno-gold labeling assay using primary antibodies raised against SYNV virion structural proteins. The presence of dense gold particles labeling the viroplasms in SYNV-GFP-M:RFP infected nuclei confirmed its identity as the authentic viral replication sites (Figure 5B). Together, the results show that in the absence of M protein, active viral replication and NC assembly result in the formation of large viroplasms. However, the mutant virus is unable to undergo virion assembly and morphogenesis.

## 4. Discussion

To obviate the antisense problem associated with the rescue of recombinant NSVs, researchers have devised a positive-strand approach to detour on assembling an agNC that can direct the synthesis of viral gRNA. Since the nascent gRNA are concomitantly encapsidated to form gNC by the NC proteins that are also expressed from the supporting plasmids, formation of dsRNA is minimized or avoided. This is critical because dsRNA formation not only presents a steric hindrance to mRNA translation and NC assembly but also incurs a potent antiviral response. We hypothesized that the steric hindrance mode might be of less concern because it is likely that only a fraction of viral mRNAs and gRNAs anneal to form dsRNAs in a crowded cellular environment. In contrast, induction of host antiviral immunity is more relevant, since a few dsRNA molecules per cell is sufficient to signal robust RNAi responses [38]. Therefore, we reasoned that if RNAi responses are thwarted by VSRs or dsRNA antagonists, the negative-strand gRNA approach may result in efficient assembly of gNC that would initiate recombinant infections on a fast track.

Using the GFP-tagged SYNV agRNA and gRNA transcription plasmids for parallel comparisons, we showed that in the presence of co-expressed VSRs, the gRNA transcripts were much more efficient than the agRNA templates in the recovery of recombinant SYNV. By diluting the agrobacterial cultures harboring the gRNA transcription plasmid, we estimated that the gRNA approach improved SYNV rescue efficiencies by about two orders of magnitude compared to the agRNA approach. The improved rescue efficiencies were manifested by much greater numbers of primary foci in infiltrated leaves, elevated systemic infection rates, and accelerated disease onset. This is unlikely due to genetic differences between recombinant SYNVs recovered from the agRNA and gRNA plasmids, because their sequences are identical and their recovered progeny virions exhibited indistinguishable infectivity and virulence upon mechanical inoculation (data now shown). Rather, the escalated systemic infection dynamics are likely a consequence of the large number of primary infection foci in agroinfiltrated leaves resulted from efficient gNC reconstitution. This notion is supported by mathematical modeling of plant virus infections showing that the number of primary infection foci positively correlates with the disease onset and efficiency of systemic infection [39,40].

Our data showed that the co-expressed VSRs are critical for SYNV recovery. In the absence of VSRs, no recombinant virus was recovered from either the infiltrated or the systemic leaves. Although a single VSR promoted SYNV rescue, simultaneous expression of multiple VSRs with different RNAi suppression mechanisms generally exhibited additive effects. We can envision two roles of the co-expressed VSRs in the gRNA-based SYNV recovery system. First, it has been shown that VSRs boost *Agrobacterium*-mediated transient gene expression [41], which would result in high levels of gRNA transcripts and NC core proteins accumulation to facilitate efficient gNC assembly. Moreover, dsRNA formation through the hybridization between viral gRNAs and mRNAs would elicit host antiviral RNAi responses, which are suppressed by the VSR proteins to promote virus rescue and efficient virus spread [42,43].

The utility of the gRNA-based rescue system is evidenced by our ability to recover SYNV-GFP-M:RFP. The matrix (M) proteins of rhabdoviruses play central roles in virion assembly and budding. An M-deficient mutant of rabies virus is severely impaired in budding, with a 500,000-fold reduction of virion production [44]. Although plant rhabdovirus M proteins have been proposed to assume similar functions [45], a deep understanding of virus budding requires generation and analysis of defined virus mutants. During the course of virion assembly and morphogenesis, SYNV M protein is postulated to condense the loosely coiled genomic RNP, leading to quenching of viral mRNA transcription and assembly of the bullet-shaped cores [46]. Subsequently, through interactions between M protein and the carboxyl-terminus of G protein, the condensed cores bud through the inner nuclear envelope to acquire the surface G protein and maturate into enveloped virus particles [47]. Here, our data showed that cells infected with SYNV-GFP-M:RFP lacked bacilliform virus particles but contained large nuclear viroplasms, which are indicative of abortive virion assembly. Our reverse genetics studies also showed that SYNV-GFP-M:RFP had a substantially reduced infectivity and a vasculature-confined tissue tropism. These phenotypes are reminiscent of and more exaggerated than those elicited by SYNV G protein deletion mutant [27], suggesting that the budding process plays important roles in systemic infection and mesophyll tissue invasion. In conclusion, a highly efficient rescue system is of critical importance for reverse genetic analyses of virus biology, especially when concerning the generation of mutant viruses with attenuated replication or virulence.

Although the general principles used for recovery of recombinant NSVs are similar, it remains to be determined whether the gRNA-based approach developed for SYNV rescue will be widely applicable to other NSVs of plants and animals. In vertebrates, dsRNA molecules are sensed by RIG-I-like immune receptors to trigger interferon responses rather than by Dicer-like enzymes to induce RNAi [48]. Consequently, vertebrate viruses have evolved various counter-defense mechanisms to antagonize the interferon-mediated antiviral immunity [49]. Prominent examples include Ebola virus VP35 and influenza virus NS1, two dsRNA binding proteins that prevent dsRNA recognition by immune receptors [35,36]. Interestingly, upon ectopic expression in plants, VP35 and NS1 proteins exhibited RNAi suppression activities through dsRNA sequestration [50,51], and our data showed that they also functioned to promote SYNV rescue. These observations raise the intriguing question of whether these proteins can overcome the antisense problem to facilitate rescue of mammalian nonsegmented NSVs from genomic cDNA copies. For segmented NSVs of plants and some animal counterparts, the current reverse genetics system schemes involve transcription of viral agRNAs that serve as templates for both genome replication and translation of NC proteins [20,21,24,29,30]. In these cases, adaptation of the gRNA approach would require additional plasmids to provide the NC proteins, and these *in trans* expressed proteins may be less efficient in recruiting the RNA templates for NC assembly [52]. Nevertheless, the unexpected high efficiency of the gRNA-based SYNV rescue system emphasizes that a similar strategy merits consideration when devising conditions for recovery of other NSVs.

## Figures and Tables

**Table 1:** Comparison of rescue efficiency of SYNV derivatives from antigenomic and genomic cDNA clones in *N. benthamiana*

Binary Plasmid ^a^	OD_600_	Foci No. ^b^	Systemic Infection ^c^
pSYNV-GFP(+)	0.2	4.3	3/45 (6.7%)
pSYNV-GFP(-)	0.2	>100	45/45 (100%)
pSYNV-GFP(-)	0.004	32.0	5/45 (11.1%)
pSYNV-GFP(-)	0.002	18.3	4/45 (8.8%)
pSYNV-GFP(-)	0.001	5.3	1/45 (2.2%)
pSYNV-GFP-M:GFP(+)	0.2	3.7	0/45 (0%)
pSYNV-GFP-M:GFP(-)	0.2	83.0	4/45 (8.8%)

^a^ Leaves of *N. benthamiana* were co-infiltrated with mixtures of *Agrobacterium* cultures harboring the plasmid encoding an SYNV antigenomic or genomic RNA derivative with the final OD_600_ indicated, the pGD-NPL plasmid for expression of the N, P, and L core proteins (final OD_600_ = 0.2), and the pGD plasmids for expression of the TEV HcPro, TBSV p19, BSMV γb viral suppressors of RNA silencing (VSRs) (final OD_600_ = 0.2). ^b^ The average numbers of infection foci in infiltrated leaves were counted at 9 dpi from three independent observation fields. ^c^ Systemic infection rates (infected/inoculated plants) were calculated at 40 dpi based on symptom observation and RT-PCR assays from three independent experiments.

## References

[1] (2004). Biology of Negative Strand RNA Viruses: The Power of Reverse Genetics.

[2] Knipe D.M., Howley P.M. (2013). Fields Virology.

[3] Lawson N.D., Stillman E.A., Whitt M.A., Rose J.K. (1995). Recombinant vesicular stomatitis viruses from DNA. Proc. Natl. Acad. Sci. USA.

[4] Whelan S.P., Ball L.A., Barr J.N., Wertz G.T. (1995). Efficient recovery of infectious vesicular stomatitis virus entirely from cDNA clones. Proc. Natl. Acad. Sci. USA.

[5] Radecke F., Spielhofer P., Schneider H., Kaelin K., Huber M., Dötsch C., Christiansen G., Billeter M.A. (1995). Rescue of measles viruses from cloned DNA. EMBO J..

[6] Roberts A., Rose J.K. (1998). Recovery of Negative-Strand RNA Viruses from Plasmid DNAs: A Positive Approach Revitalizes a Negative Field. Virology.

[7] Schnell M., Mebatsion T., Conzelmann K. (1994). Infectious rabies viruses from cloned cDNA. EMBO J..

[8] Conzelmann K.K. (2004). Reverse Genetics of Mononegavirales. Curr. Top. Microbiol. Immunol..

[9] Rose J.K. (1996). Positive strands to the rescue again: A segmented negative-strand RNA virus derived from cloned cDNAs. Proc. Natl. Acad. Sci. USA.

[10] Hur S. (2019). Double-Stranded RNA Sensors and Modulators in Innate Immunity. Annu. Rev. Immunol..

[11] Ding S.-W. (2010). RNA-based antiviral immunity. Nat. Rev. Immunol..

[12] Collins P.L., Mink M.A., Stec D.S. (1991). Rescue of synthetic analogs of respiratory syncytial virus genomic RNA and effect of truncations and mutations on the expression of a foreign reporter gene. Proc. Natl. Acad. Sci. USA.

[13] Garcin D., Pelet T., Calain P., Roux L., Curran J., Kolakofsky D. (1995). A highly recombinogenic system for the recovery of infectious Sendai paramyxovirus from cDNA: Generation of a novel copy-back nondefective interfering virus. EMBO J..

[14] Hoffman M.A., Banerjee A.K. (1997). An infectious clone of human parainfluenza virus type 3. J. Virol..

[15] Durbin A.P., Hall S.L., Siew J.W., Whitehead S., Collins P.L., Murphy B.R. (1997). Recovery of Infectious Human Parainfluenza Virus Type 3 from cDNA. Virology.

[16] Volchkov V.E., Volchkova V.A., Mühlberger E., Kolesnikova L.V., Weik M., Dolnik O., Klenk H.-D. (2001). Recovery of Infectious Ebola Virus from Complementary DNA: RNA Editing of the GP Gene and Viral Cytotoxicity. Science.

[17] Schneider U., Schwemmle M., Staeheli P. (2005). Genome trimming: A unique strategy for replication control employed by Borna disease virus. Proc. Natl. Acad. Sci. USA.

[18] Sanchez A.B., de la Torre J.C. (2006). Rescue of the prototypic Arenavirus LCMV entirely from plasmid. Virology.

[19] Bridgen A., Elliott R.M. (1996). Rescue of a segmented negative-strand RNA virus entirely from cloned complementary DNAs. Proc. Natl. Acad. Sci. USA.

[20] Blakqori G., Weber F. (2005). Efficient cDNA-based rescue of La Crosse bunyaviruses expressing or lacking the nonstructural protein NSs. J. Virol..

[21] Lowen A.C., Noonan C., McLees A., Elliott R.M. (2004). Efficient bunyavirus rescue from cloned cDNA. Virology.

[22] Billecocq A., Gauliard N., Le May N., Elliott R.M., Flick R., Bouloy M. (2008). RNA polymerase I-mediated expression of viral RNA for the rescue of infectious virulent and avirulent Rift Valley fever viruses. Virology.

[23] Habjan M., Penski N., Spiegel M., Weber F. (2008). T7 RNA polymerase-dependent and -independent systems for cDNA-based rescue of Rift Valley fever virus. J. Gen. Virol..

[24] Ikegami T., Won S., Peters C.J., Makino S. (2006). Rescue of Infectious Rift Valley Fever Virus Entirely from cDNA, Analysis of Virus Lacking the NSs Gene, and Expression of a Foreign Gene. J. Virol..

[25] Kato A., Sakai Y., Shioda T., Kondo T., Nakanishi M., Nagai Y. (1996). Initiation of Sendai virus multiplication from transfected cDNA or RNA with negative or positive sense. Genes Cells.

[26] Jackson A.O., Li Z. (2016). Developments in Plant Negative-Strand RNA Virus Reverse Genetics. Annu. Rev. Phytopathol..

[27] Wang Q., Ma X., Qian S., Zhou X., Sun K., Chen X., Zhou X., Jackson A.O., Li Z. (2015). Rescue of a Plant Negative-Strand RNA Virus from Cloned cDNA: Insights into Enveloped Plant Virus Movement and Morphogenesis. PLoS Pathog..

[28] Gao Q., Xu W., Yan T., Fang X., Cao Q., Zhang Z., Ding Z., Wang Y., Wang X.-B. (2019). Rescue of a plant cytorhabdovirus as versatile expression platforms for planthopper and cereal genomic studies. New Phytol..

[29] Feng M., Cheng R., Chen M., Guo R., Li L., Feng Z., Wu J., Xie L., Hong J., Zhang Z. (2019). Rescue of tomato spotted wilt virus entirely from complementary DNA clones. Proc. Natl. Acad. Sci. USA.

[30] Verchot J., Herath V., Urrutia C.D., Gayral M., Lyle K., Shires M.K., Ong K., Byrne D. (2020). Development of a reverse genetic system for studying rose rosette vrus in whole plants. Mol. Plant-Microbe Interact..

[31] Ganesan U., Bragg J.N., Deng M., Marr S., Lee M.Y., Qian S., Shi M., Kappel J., Peters C., Lee Y. (2013). Construction of a Sonchus Yellow Net Virus Minireplicon: A Step toward Reverse Genetic Analysis of Plant Negative-Strand RNA Viruses. J. Virol..

[32] Goodin M.M., Dietzgen R.G., Schichnes D., Ruzin S., Jackson A.O. (2002). pGD vectors: Versatile tools for the expression of green and red fluorescent protein fusions in agroinfiltrated plant leaves. Plant J..

[33] Jackson A., Christie S. (1977). Purification and some physicochemical properties of sonchus yellow net virus. Virology.

[34] Li W.X. (2002). Induction and Suppression of RNA Silencing by an Animal Virus. Science.

[35] Prins K.C., Delpeut S., Leung D.W., Reynard O., Volchkova V.A., Reid S.P., Ramanan P., Cárdenas W.B., Amarasinghe G.K., Volchkov V.E. (2010). Mutations Abrogating VP35 Interaction with Double-Stranded RNA Render Ebola Virus Avirulent in Guinea Pigs. J. Virol..

[36] Garcia-Sarstre A., Egorov A., Matassova D., Brandtbc S., Levy D.E., Durbin J.E., Palese P., Musterbc T. (1998). Influenza A Virus Lacking the NS1 Gene Replicates in Interferon-Deficient Systems. Virology.

[37] Okumura A., Harty R.N. (2011). Rabies Virus Assembly and Budding. Adv. Clin. Chem..

[38] Fire A., Xu S., Montgomery M.K., Kostas S.A., Driver S.E., Mello C.C. (1998). Potent and specific genetic interference by double-stranded RNA in Caenorhabditis elegans. Nature.

[39] Lafforgue G., Tromas N., Elena S.F., Zwart M.P. (2012). Dynamics of the Establishment of Systemic Potyvirus Infection: Independent yet Cumulative Action of Primary Infection Sites. J. Virol..

[40] Rodrigo G., Zwart M.P., Elena S.F. (2014). Onset of virus systemic infection in plants is determined by speed of cell-to-cell movement and number of primary infection foci. J. R. Soc. Interface.

[41] Saxena P., Hsieh Y.-C., Moreau M., Sainsbury F., Saunders K., Lomonossoff G.P., Scholthof H.B. (2010). Improved foreign gene expression in plants using a virus-encoded suppressor of RNA silencing modified to be developmentally harmless. Plant Biotechnol. J..

[42] Kasschau K.D., Carrington J.C. (2001). Long-Distance Movement and Replication Maintenance Functions Correlate with Silencing Suppression Activity of Potyviral HC-Pro. Virology.

[43] Qu F., Morris T.J. (2002). Efficient Infection of Nicotiana benthamiana by Tomato bushy stunt virus Is Facilitated by the Coat Protein and Maintained by p19 Through Suppression of Gene Silencing. Mol. Plant-Microbe Interact..

[44] Mebatsion T., Weiland F., Conzelmann K.K. (1999). Matrix Protein of Rabies Virus Is Responsible for the Assembly and Budding of Bullet-Shaped Particles and Interacts with the Transmembrane Spike Glycoprotein G. J. Virol..

[45] Jackson A.O., Dietzgen R.G., Goodin M., Bragg J.N., Deng M. (2005). Biology of Plant Rhabdoviruses. Annu. Rev. Phytopathol..

[46] Zhou X., Sun K., Zhou X., Jackson A.O., Li Z. (2019). The Matrix Protein of a Plant Rhabdovirus Mediates Superinfection Exclusion by Inhibiting Viral Transcription. J. Virol..

[47] Sun K., Zhou X., Lin W., Zhou X., Jackson A.O., Li Z. (2018). Matrix-glycoprotein interactions required for budding of a plant nucleorhabdovirus and induction of inner nuclear membrane invagination. Mol. Plant Pathol..

[48] Cullen B.R., Cherry S., Tenoever B.R. (2013). Is RNA Interference a Physiologically Relevant Innate Antiviral Immune Response in Mammals?. Cell Host Microbe.

[49] Tenoever B.R. (2016). The Evolution of Antiviral Defense Systems. Cell Host Microbe.

[50] Bucher E., Hemmes H., de Haan P., Goldbach R., Prins M. (2004). The influenza A virus NS1 protein binds small interfering RNAs and suppresses RNA silencing in plants. J. Gen. Virol..

[51] Haasnoot J., de Vries W., Geutjes E.-J., Prins M., de Haan P., Berkhout B. (2007). The Ebola Virus VP35 Protein Is a Suppressor of RNA Silencing. PLoS Pathog..

[52] Qian S., Chen X., Sun K., Zhang Y., Li Z. (2017). Capped antigenomic RNA transcript facilitates rescue of a plant rhabdovirus. Virol. J..

